# Proteins Involved in Distinct Phases of Cold Hardening Process in Frost Resistant Winter Barley (*Hordeum vulgare* L.) cv Luxor

**DOI:** 10.3390/ijms14048000

**Published:** 2013-04-12

**Authors:** Iva Hlaváčková, Pavel Vítámvás, Jiří Šantrůček, Klára Kosová, Sylva Zelenková, Ilja Tom Prášil, Jaroslava Ovesná, Radovan Hynek, Milan Kodíček

**Affiliations:** 1 Department of Biochemistry and Microbiology, Institute of Chemical Technology Prague, Technická 5, 166 28 Prague 6, Czech Republic; E-Mails: santrucj@vscht.cz (J.Š.); hynekr@vscht.cz (R.H.); kodicekm@vscht.cz (M.K.); 2 Department of Genetics and Plant Breeding, Crop Research Institute, Drnovská 507/73, 161 06 Prague 6, Czech Republic; E-Mails: vitamvas@vurv.cz (P.V.); kosova@vurv.cz (K.K.); prasil@vurv.cz (I.T.P.); ovesna@vurv.cz (J.O.); 3 Department of Plant Experimental Biology, Charles University in Prague, Albertov 6, 128 43 Prague 2, Czech Republic; E-Mail: zelen@natur.cuni.cz

**Keywords:** cold shock, cold and frost acclimation, winter hardiness, hardening, chloroplasts, 2D-DIGE, peptide-mapping

## Abstract

Winter barley is an economically important cereal crop grown in higher latitudes and altitudes where low temperatures represent an important environmental constraint limiting crop productivity. In this study changes in proteome of leaves and crowns in a frost tolerant winter barley cv. Luxor in relation to short and long term periods of cold followed by a brief frost treatment were studied in order to disclose proteins responsible for the cold hardening process in distinct plant tissues. The mentioned changes have been monitored using two dimensional difference gel electrophoresis (2D-DIGE) with subsequent peptide-mapping protein identification. Regarding approximately 600–700 distinct protein spots detected on 2D gels, there has been found at least a two-fold change after exposure to low temperatures in about 10% of proteins in leaves and 13% of proteins in crowns. Protein and nitrogen metabolic processes have been influenced by low temperature to a similar extent in both tissues while catabolism, carbohydrate metabolism and proteins involved in stress response have been more affected in crowns than in leaves. The range of changes in protein abundance was generally higher in leaves and chloroplast proteins were frequently affected which suggests a priority to protect photosynthetic apparatus. Overall, our data proved existence of slightly different response strategies to low temperature stress in crowns and leaves, *i.e.*, tissues with different biological role. Moreover, there have been found several proteins with large increase in accumulation, e.g., 33 kDa oxygen evolving protein of photosystem II in leaves and “enhanced disease susceptibility 1” in crowns; these proteins might have potential to indicate an enhanced level of frost tolerance in barley.

## 1. Introduction

Barley can be cultivated under harsher conditions than many other crops, mainly due to its high tolerance to drought and low temperatures [1]. Comprehension of mechanisms by which barley copes with cold and frost is essential for breeding new tolerant cultivars and thus for increasing yields in areas of low production.

Like other plants from the tribe *Triticeae*, barley exists in spring and winter varieties. For winter cultivars, grown in areas where the temperatures during winter drop below zero, the high frost tolerance is crucial. Moreover, these cultivars require external signal for transition from vegetative phase into reproductive phase; this signal can be vernalization or length of photoperiod [2]. The maximum frost tolerance is reached during the vegetative phase of growth by the cold hardening process (cold acclimation), which takes place predominantly in autumn [3]. The first stage of cold hardening begins at low temperatures above 0 °C, while the second stage requires considerably lower but still non-lethal temperatures [4]. Cold hardening leads to alterations in plasma membrane composition, changes in carbohydrate metabolism and secondary metabolism, photosynthesis, enhanced detoxification and oxido-reduction processes, increased production of osmolytes and synthesis of stress-related proteins [5]. Among the latter, heat shock proteins (HSP) and late embryogenesis abundant (LEA) proteins represent a major class of protective molecules [6]. Proteomic studies concerning abiotic stresses in plants were recently summarized [7]; at low temperatures, an increase in accumulation of glycolytic enzymes, components of photosynthetic electron transport chain, cp29 RNA-binding protein, reactive oxygen species scavenging enzymes, LEA proteins, chaperones and 70 kDa heat shock protein (HSP70) has been observed repeatedly while accumulation of enzymes involved in saccharide anabolism was decreased. Only few proteomic studies dealt with low temperatures effect on plants belonging to tribe *Triticeae*; in all of them, wheat was used as a model [8–11].

Our knowledge of cold hardening processes in barley is to date based mainly on transcriptomic studies and studies using one dimensional polyacrylamide gel electrophoresis. Among the best characterized cold induced genes, there belong *Wcs120*[12] and *DHN5*[7,13]; proteins encoded by these genes might serve as frost tolerance markers [14,15]. It was shown that majority of cold regulated genes in barley reacted as early as short period of cold (1 day) occurred and only a limited number of barley genes displayed lasting response to prolonged cold treatment [16]. About 67% of genes responsive to cold were found to be chloroplast-dependent cold-regulated genes in leaf tissues. Thus the chloroplasts were suggested to play a major role in the control of barley molecular adaptation to cold [17] and they were proposed as a target for crop improvement [18]. The link between chloroplast activity and the susceptibility of the plant to stress together with retrograde plastid-to-nucleus signaling was demonstrated in *Arabidopsis thaliana*[19].

In winter cereals, it has been shown that survival of a plant depends on the survival of its crown tissues [20–22]. This part of a plant connects roots and stem and contains meristematic tissue which is responsible for growth restoration in the spring. Recently, different transcription patterns of genes involved in pathways responsible for osmolyte production (sucrose, raffinose, γ-aminobutyric acid), sugar signaling (trehalose metabolism) and secondary metabolism (lignin synthesis) were observed in barley leaves and crowns at low temperatures [23].

In our study, response of barley leaves and crowns to low temperatures (1 day cold shock, 21 days cold acclimation, 1 day frost shock) has been studied by 2D-DIGE coupled with peptide fingerprinting protein identification method using MALDI-TOF mass spectrometry. The aim of this study was to characterize processes leading to enhanced winter hardiness in distinct tissues at proteome level.

## 2. Results and Discussion

### 2.1. Proteomic Analysis

**Figure 1:**
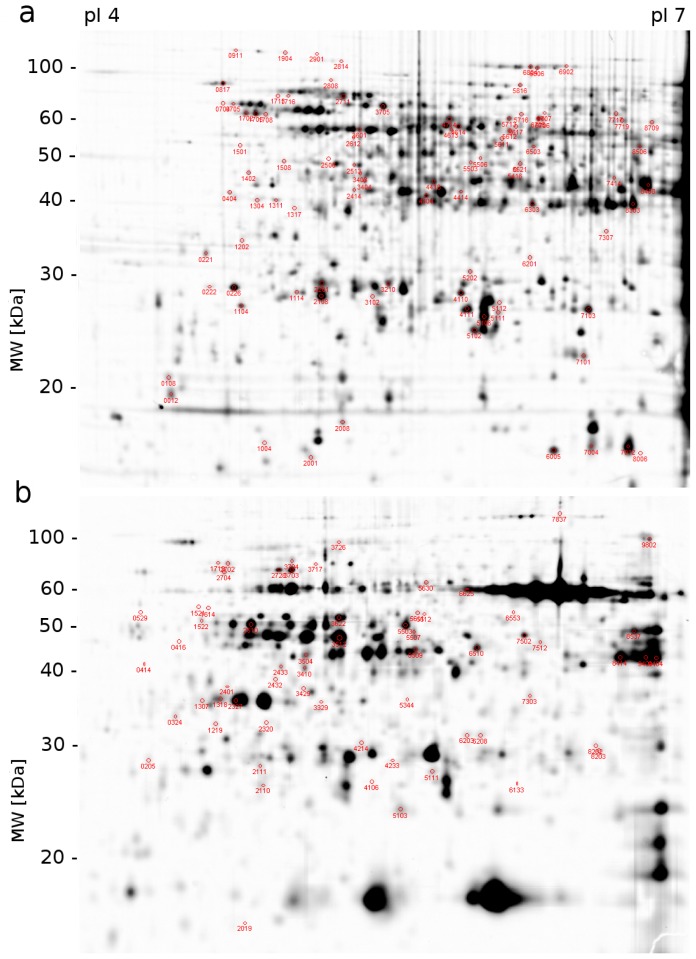
Representative proteome maps of 50 μg total proteins from (**a**) crowns and (**b**) leaves of barley (*Hordeum vulgare* L.) separated by two dimensional difference gel electrophoresis (2D-DIGE) (24 cm immobilised pH gradient (IPG) strip, pI 4–7, 1 mm thick gel). At least two times up- or down-accumulated proteins (*p* = 0.05, *t*-test, *n* = 3) are marked with the corresponding spot numbers.

Seedlings of winter barley were exposed to “cold shock” (1 day of 3 °C), “cold acclimation” (21 days of 3 °C) and “frost shock” (1 day of −3 °C after, cold acclimation) and the proteins extracted from leaves and crowns were separated using 2D-DIGE (Figure S1 in Supplemental data). On each gel, approximately 1200 protein spots were distinguished (Figure 1) and with regard to gel to gel variance, it was possible to quantify approximately 700 proteins in crowns and 600 proteins in leaves. The lower number of proteins quantified in leaves could be explained by the presence of highly abundant chloroplast proteins (such as Rubisco large and small chains, see Figure 1b—approximately 60 kDa and 20 kDa) in leaves which decreases the possibility to detect low abundant proteins. This difficulty can be solved by Rubisco depletion; however, each manipulation with samples brings also artificial changes in proteins abundance levels. Therefore, the use of non-photosyntetic tissues, such as crowns in cereals, for gel based proteomic approaches can provide a better view on behavior of low abundant proteins that are not detectable in leaf samples.

In total, 90 proteins in crowns and 63 proteins in leaves changed their abundance at least twice in consequence of cold and frost treatment according to Student’s *t*-test (*p* = 0.05, three biological replicates). By employing the peptide fingerprinting method, 48 proteins in crowns and 39 proteins in leaves were identified (Tables 1 and 2, Table S1 in Supplementary data); among them several proteins with documented relation to cold or freezing stress were found, e.g., (HSP70) and chaperonins [24,25]. In order to facilitate comparison with transcriptome studies, corresponding probe set identifiers on Barley 1 Affymetrix chip [16,17,23] have been found for 8 identified proteins (Tables 1 and 2, Table S1 in Supplementary data). Several proteins (e.g., fructokinase in spot n. 1304, 1311) were identified more than once in the same tissue; these repeatedly observed proteins differed in molecular mass and/or pI observed on gel which is likely due to different isoforms of the same protein, protein modifications or intracellular proteolysis. Some of the differently accumulated proteins were not identified as their abundances on gel were not high enough for subsequent peptide mapping (the peptide mass spectra of appropriate quality were not obtained or the peptide mass spectra did not match to any protein in the database).

Only one protein was identified in samples from both tissues—glutamine synthetase (gi|755762) with almost similar decrease of abundance after cold in both tissues (spot n. 6806 in crowns and 2510 in leaves); moreover, the glutamine synthetase GSr1 (gi|40317416) isoform in crowns also decreased its abundance.

In total, five members of different protein families have been identified in both tissues; AAA ATPase (gi|255070529 in leaves and gi|194707130 in crowns), ribosomal proteins (60S in leaves—gi|225465175; 40S in crowns—gi|357113738), elongation factor Tu (gi|226508704 in leaves, gi|326514754 and gi|357149925 in crowns), vacuolar proton ATPase (gi|145351306 in leaves, gi|11527563 in crowns) and chaperonin 60 kDa (gi|255560267 in leaves, gi|326533328 in crowns). The noticeable difference between leaf and crown tissue was observed in protein spot 4106 identified as AAA ATPase on leaf gels. Spot 4106 increased its abundance after cold shock and decreased its abundance after cold acclimation in leaves whereas in crowns the abundance of AAA ATPase was constantly decreasing in course of low temperature exposure. The difference was also found in chaperonin 60 kDa; there was an increase in its abundance after cold acclimation in leaves (gi|255560267) whereas in crowns, its abundance was decreased (gi|326533328) at the same time. Different changes in protein abundance of AAA ATPase and chaperonin 60 kDa could be explained by identification of different isoforms with probably slightly different activity under cold stress. However, most of the proteins identified in both tissues showed similar changes in abundance under the cold treatment and this result thus may indicate to some extent analogous regulation of common cold-responsive pathway in cold stressed plant tissues (such as increased dehydrin levels in cold-treated leaves and crowns; [26]).

### 2.2. Biological Processes Influenced by Low Temperatures in Leaves and Crowns

**Figure 2:**
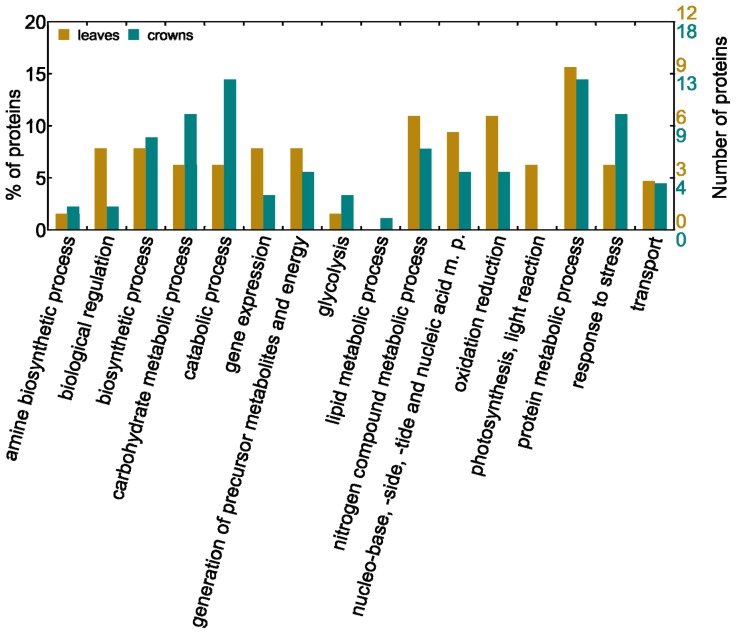
Functional classification of differently accumulated proteins showing a comparison between crowns and leaves of winter barley exposed to cold and frost. In total, 48 proteins in crowns and 39 proteins in leaves were assigned to one or more of 16 categories selected for classification using Web Gene Ontology Annotation Plotting (WEGO) application. On the y axis the percentage of differently accumulated proteins possesing similar biological function in distinct tissues can be seen, on the x axis respective biological processes are listed. WEGO classification reflects the fact that single protein can be involved in several processes, thus sum of % in all categories is higher than 100.

Biological functions of differently accumulated proteins were determined by their GO annotation in domain of biological processes according to UniProtKB (Tables 1 and 2, Table S1 in Supplementary data). In total, 19 proteins (22% of identified proteins) were assigned to category “Unknown” as their functions have not been determined yet. However, this result indicates that these yet “unknown” proteins are cold-regulated (14 proteins revealed a decreased accumulation and 5 proteins revealed an increased accumulation during cold treatment). The global overview of biological processes affected by low temperatures was obtained using WEGO application; the functional classification using WEGO reflects the fact that a single protein can be involved in various processes and therefore classified in more than one functional category. After setting of GO annotation level to five, 16 functional categories were chosen to cover our data and to pointed out existing differences between leaves and crowns in response to low temperatures (Figure 2, Tables S1 and S2 in Supplementary data).

The “protein metabolic processes” represents the largest functional category being formed by approximately 15% of identified proteins both in leaves and crowns. Regulation of protein metabolism; represented by proteases, elongation factors and ribosomal proteins, appears to be of the same significance in leaves and crowns in the response to low temperatures. Number of proteins in this category suggests importance of unfunctional proteins removal and necessity of proteosynthesis regulation. The second largest category “catabolic processes” (6% of identified differently accumulated proteins in leaves and 14% in crowns) confirmed previous finding that the cold hardening is an active and energy demanding process [27]. The next large category, “nitrogen compound metabolism”, was influenced to similar extent in both tissues (11% of identified proteins in leaves and 8% in crowns, e.g., glutamine synthetase) thus implying universal importance of these proteins in stress response to cold. Changes in nitrogen metabolism related proteins accumulation have been so far observed mainly as a consequence of drought and salinity stress [7]. Other noteworthy functional categories contained proteins involved in “carbohydrate metabolism” (6% of identified proteins in leaves and 11% in crowns, e.g., pyruvate kinase) and “response to stress” (6% of identified proteins in leaves and 11% in crowns, e.g., peroxygenase). Interestingly, protein grouped into category “lipid metabolic process” (*i.e.*, lipase “enhanced disease susceptibility 1”—EDS1; gi|149939605, Table S1) was found only in crowns. In this case, it is more probable that accumulation of this protein has also been changed in leaves but these changes were undetectable due to presence of highly abundant photosynthetic proteins in leaves (see subchapter 2.1.). It is not surprising that proteins (33 kDa oxygen evolving protein of photosystem II (PsbO), gi|115436780; chlorophyll a-b binding proteins, gi|115778 and gi|115793; Table S1) grouped into “photosynthesis, light reaction” functional category have been found only in leaves and not in crowns which are non-photosynthetic tissue.

On account of the differences found in numbers and quality of proteins responsible for separate biological processes influenced by low temperatures in distinct tissues, it is reasonable to believe in existence of slightly different response strategies to low temperature stress in crowns (plant survival and growth renewal) and leaves (energy metabolism), *i.e.*, tissues with different biological role [20].

### 2.3. Proteins with Large Changes in Abundance

Exploratory data analysis revealed outlying proteins in our data sets, the proteins which changed their abundance excessively compared to the rest of differently accumulated proteins. On the basis of the large changes in accumulation of these proteins, we can conclude that they may play substantial roles in frost tolerant winter barley response to low temperature. Since the data sets observed in leaves and crowns differed in their characteristics, the extent of changes in proteins abundances in leaves were greater compared to crowns (Figure S2 and Table S3 in Supplementary data), the limit for outliers (the proteins which displayed excessive changes in abundance) has been set as different values for leaves and crowns, respectively. The boxplot method (2.698 σ) suggested 8 outliers in leaves which changed their abundance at least 8 times and 7 outliers in crowns which changed abundance at least 6 times (Tables 1 and 2, in more detail Tables S1 and S3 in Supplementary data).

**Figure 3:**
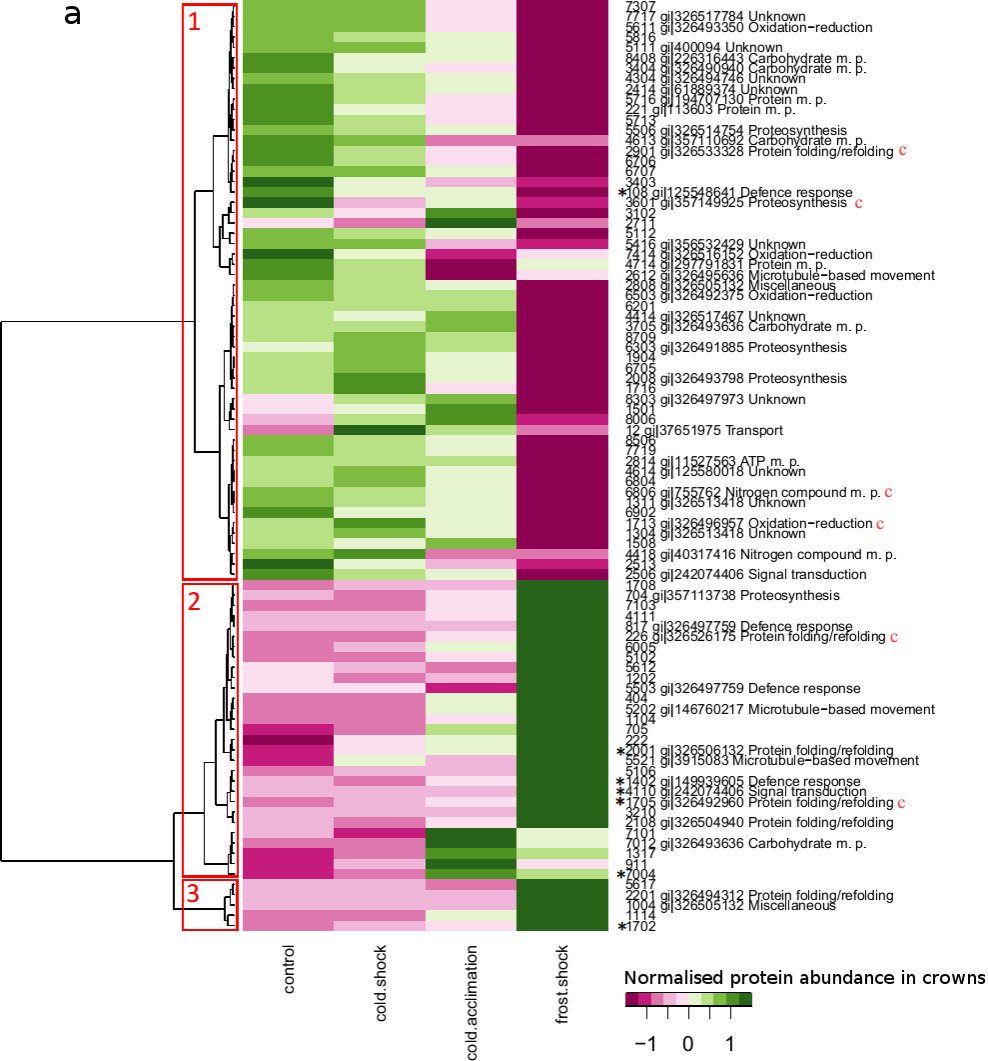

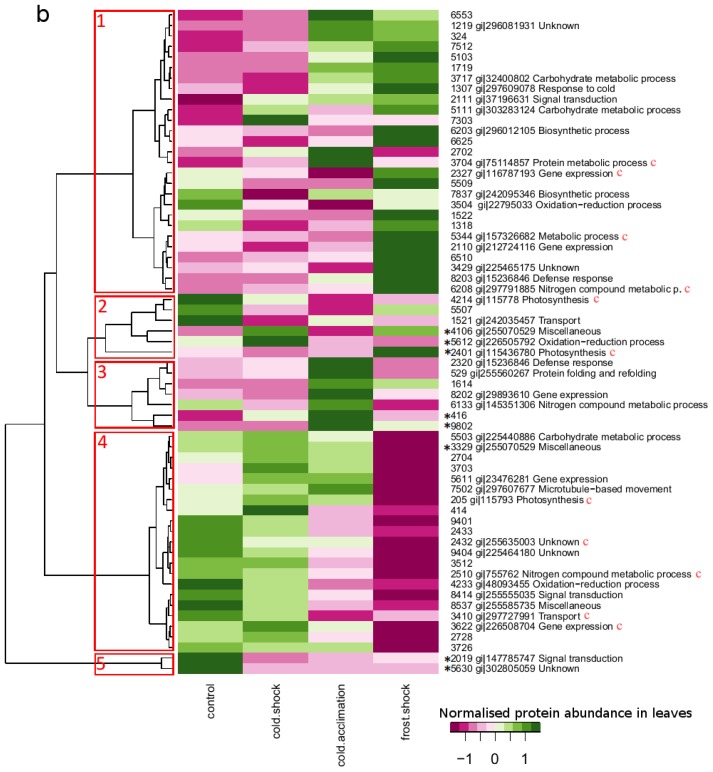
Hierarchical cluster analysis of proteins differently accumulated in (**a**) crowns and (**b**) in leaves in course of low temperature exposure. Red boxes mark clusters of proteins which have similar mode of accumulation; dendrogram was constructed using Ward’s method and cut off at height *h* = 10. The sign * marks proteins which changed their abundance at least 8 times in leaves and 6 times in crowns, letter “c” marks proteins from chloroplasts.

In leaves, the outliers were found in all clusters (see subchapter 2.4., Figure 3, Table 2) except Cluster 1 (increase of abundance) and only 5 of the 8 identified proteins have known function (Table 2, Table S1 in Supplementary data). Among them, there was AAA ATPase (gi|255070529) which was found in two spots; in spot 4106, its abundance was 10 times increased after frost shock/cold acclimation while in spot 3329, the abundance was 9 times decreased (Table S1 in Supplementary data). According to position on gel, these two spots differed slightly in molecular weight and pI (spot 4106: 117 kDa, pI 5.37 and spot 3329: 112 kDa, pI 5.6), these differences might be caused by protein modifications in reaction to frost stress. Proteins of AAA family are essential for many cellular functions including degradation of misfolded proteins; previously they have been observed as up-accumulated during cold acclimation [28]. Another highly accumulated protein in leaves was protein PsbO (gi|115436780, 19 times increase after frost shock/cold shock), a part of the oxygen-evolving complex which stabilises manganese. Together with the gene for chloroplast protein PsbO several other genes are co-expressed, including ATPases [29], which is in accordance with our finding. The enzyme UDP-glucose 6-dehydrogenase (gi|226505792), which is involved in diverting UDP-Glc to cell wall biosynthesis among other processes, increased abundance twice after cold shock and then excessively decreased abundance (10 times decreased after frost shock/cold shock). Low temperature stress leads to cell dehydration and causes changes in cell volume and cell compartment organization; therefore, the adjustment of cell wall is a necessity for holding cell integrity; several enzymes participating in UDP-Glc formation and thus cell wall biogenesis have been already observed as up-accumulated after short cold treatments, e.g., UDP-glucose pyrophosphorylase or sucrose synthase 1 [30]. In leaves, the hypothetical protein VITISV_033291” (gi|147785747) was 11 times decreased after cold shock and then 3 times increased both after cold acclimation and frost shock. This protein belongs to protein phosphatase family C (PP2C), one of the largest families in the plant kingdom, and proteins from this family have been found as regulators of signal transduction pathways and also involved in development [31]. It has been shown by antisense approaches that down-regulation of PP2C accelerated plant development and increased freezing tolerance [32]; we observed a similar trend at protein level in our experiment.

Crown outliers were found in all clusters but most of them belonged to Cluster 2; 5 out of 7 outliers were identified. Amongst the identified proteins with known function, there has been found chloroplast HSP70 (gi|326492960, 7 times increased after frost shock) which is a molecular chaperone involved in protein aggregation prevention and refolding under normal and stress conditions. It also takes part in protein import, translocation and degradation [33]. Another protein with outlying changes in abundance in crowns was EDS1 (gi|149939605, 7 times increased after frost shock compared to cold shock) which controls, among other processes, cell death propagation in response to chloroplast-derived reactive oxygen species [34]. The next two outliers identified in crowns (hypothetical protein OsI_16233 and hypothetical protein SORBIDRAFT_06g029250) are according to GO annotation involved in defense response; the last identified protein (predicted protein gi|326505132) has not been assigned to any function yet.

Interestingly, the extent of changes in protein abundance and the occurrence of outliers were generally higher in leaves, implying a need of an increased level of proteosynthesis and protein degradation during cold acclimation. The fact that the numbers of all differently accumulated proteins were higher in crowns (Table 1) corresponded to the importance of crowns for plant survival [20]. Moreover, outliers found in leaves showed large increase (e.g., 33 kDa oxygen evolving protein of photosystem II) or decrease (e.g., UDP-glucose 6-dehydrogenase) in abundance mainly after cold acclimation and frost shock and they belonged mostly to Cluster 2 and 3 whereas in crowns most of outliers mounted their abundance after frost shock (e.g., EDS1) and they were sorted to Cluster 3. This implies that proteins with large changes in abundance were highly accumulated under cold treatment in crowns (e.g., HSP70, EDS1) contrary to high decrease of some proteins accumulation in leaves (e.g., PsbO, UDP-glucose 6-dehydrogenase, AAA ATPase) in at least one sampling time during cold treatment. From our previous studies on dehydrins, e.g., [7], it is known that crop winter cultivars accumulate some protective compounds such as dehydrins also under optimal temperature of 20 °C and these proteins are accumulated in higher levels in crowns than in leaves [26]. On the other hand, leaves as photosynthetic organs play an important role in an energy-demanding process of cold-acclimation which is needed for plant survival at low temperatures. Therefore, our results indicated that after sensing cold the leaf cells reorganized some of their anabolic and catabolic processes to a higher extent when compared to crown cells; these large changes might be in order to protect photosynthetic apparatus.

The large changes in protein abundance after cold treatment observed at outliers could also indicate a potential of these proteins to evaluate cereal cold tolerance (such as dehydrins, e.g., [35]). However, these cold-regulated proteins should be first tested in a large set of differently cold-tolerant barley genotypes to verify them as possible protein markers.

### 2.4. Protein Accumulation Modes Induced by Low Temperature

**Figure 4:**
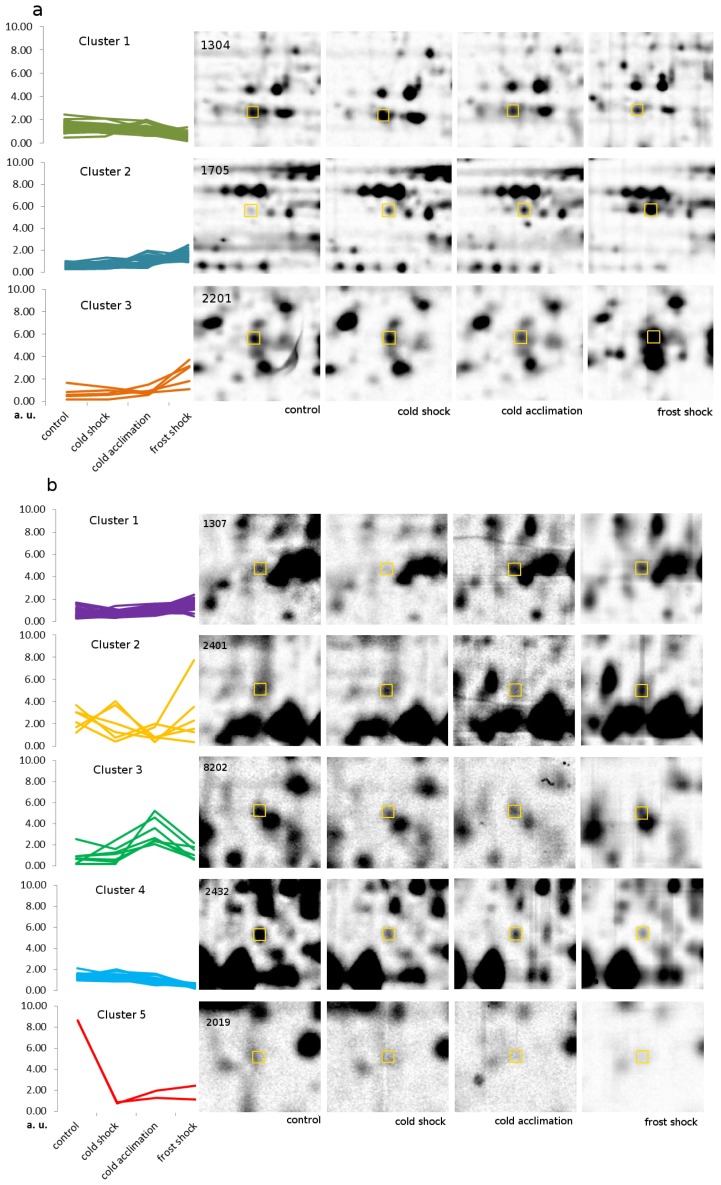
Modes of protein accumulation in (**a**) crowns and (**b**) leaves after low temperature exposure. Vertical axis represents protein abundance and horizontal axis represents different treatments, all proteins in the same cluster are drawn in the same colour. Representative protein spots pictures on 2D-gels for different treatments are given for each cluster; spot number is in left upper corner of the image and corresponding proteins are listed in Tables 1 and 2.

Hierarchical cluster analysis sorted differently accumulated proteins to three clusters in crowns and five clusters in leaves (Tables 1 and 2, Figures 3 and 4, Table S1 in Supplementary data). Proteins of which abundances were predominantly increasing in course of low temperature exposure were found in Cluster 1 in leaves (27 proteins/15 identified) and Cluster 2 in crowns (29 proteins/12 identified), the continuously mounting abundance suggests their importance in all phases of cold hardening process. Among these proteins, there are, e.g., HSP70 in crowns and ATP-dependent zinc metalloprotease FTSH 2 in leaves with documented relation to cold stress [6,28].

In Cluster 4 in leaves (21 proteins/13 identified) and in Cluster 1 in crowns (56 proteins/34 identified), there are proteins of which abundances decreased gradually after low temperature exposure, e.g., 23 kDa jasmonate-induced protein (JIP-23) in crowns (gi|400094, Contig1675_s_at); JIP-23 might regulate cell wall polysaccharide synthesis [36]. In barley leaves, JIP-23 genes were expressed upon various stresses such as drought, sorbitol treatment and cold [16,37]. However, transcript (Contig1675_s_at) of the gene belonging to this protein displayed downregulation after prolonged cold treatment (4 °C, 49 day) in barley [16]. Proteins of which abundances have decreased upon cold both in leaves and in crowns include elongation factor Tu and glutamine synthetase (GS1 and GS2). Elongation factor Tu plays a key role in the elongation phase of protein synthesis in plant organelles including mitochondria and plastids and has an important role in stress responses; it has been reported in a transcriptomic study [38] as up-accumulated after cold stress 6/2 °C and at proteomic level as up-accumulated after cold stress 15 °C, but no up-accumulation was observed after cold stress of 5 °C [30], which is in agreement with our results. Glutamine synthetase is involved in primary nitrogen assimilation and glutamine generation (isoenzyme form GS1), as well as in NH_4_^+^ reassimilation released via photorespiration (isoenzyme form GS2) [39]. Glutamine synthetase has been observed to be up-accumulated during salt stress [40,41]; nevertheless, from overexpression experiment [42] it can be concluded that lower abundance of GS1 might have a positive influence on frost resistance. Furthermore, it can be seen that in crowns the key proteins of nitrogen metabolism (glutamine synthetases GS1 and GS2) and almost all proteins related to carbohydrate metabolism (glycolysis and pentose-phosphate shunt) are placed in the Cluster 1, thus they are gradually downregulated in a similar manner. The exception is spot 7012—enolase, which mounted its abundance after cold treatment, but according to its molecular weight observed on gel (Table S1 in Supplementary data), it is most probably only a fragment after degradation. Interestingly, C2 domain-containing protein (Cluster 1; gi|61889374, gradually decreased after cold) has been observed on transcriptomic level (Contig112_at) as upregulated after cold shock in barley seedlings (4 °C, 1 day; [16]). This discrepancy is an illustration of the problematic comparison of transcriptomic results and proteomic results. Generally, the changes in abundance of stress-regulated transcripts between control and stress-treated samples are higher than similar changes observed at protein level (e.g., JIP-23 in [16]). The accumulation of transcript in tissue does not necessarily indicate real abundance of the corresponding protein since there are several levels of translational regulation, the translated proteins can be maintained for a long time (such as dehydrins under cold, e.g., [13,26] or degraded in a short time. Therefore, the changes in gene expression at transcript level do not often correspond with the changes at protein level [7].

Cluster 5 in leaves (2 proteins/2 identified) contains proteins of which abundances were strongly decreased after cold shock and then slightly increased again after cold acclimation; one of the identified proteins was “hypothetical protein VITISV_033291” (gi|147785747) which showed large decrease in its abundance.

Conspicuous changes in abundance induced by either cold shock or frost shock were exhibited mainly by proteins in Cluster 2 in leaves (6 proteins/5 identified), these proteins therefore seem to be of importance during sudden short low temperature stress, e.g., AAA ATPase and chlorophyll a-b binding protein 1 observed previously in [28,43].

Proteins of which abundances increased exclusively after cold acclimation were found in Cluster 3 in leaves (7 proteins/4 identified), e.g., chaperonin 60 kDa, peroxygenase 1. Peroxygenases are enzymes involved in the biosynthesis of phytooxylipins, which play important roles in plant defense responses [44]. Similarly, Cluster 3 in crowns (5 proteins/2 identified) consists of proteins which did not change their abundance after cold shock noticeably but their abundance increased significantly after cold acclimation or frost shock, thus they are likely of considerable role in later phases of cold hardening process. Two identified proteins were “predicted protein” gi|326494312 which is a chaperon responsible for protein folding and “predicted protein” gi|326505132 from protein family von Willebrand factor, type A with RNA-binding activity. Recently, protein LGD1 which possesses von Willebrand factor type A (vWA) domain has been studied in rice. Isoforms of this protein were detected mainly in nucleus and partly in cytoplasm and it was demonstrated that LGD1 regulates rice vegetative growth and development [45].

### 2.5. Importance of Chloroplasts in the Cold Hardening Process

Cellular localization of identified differently accumulated proteins was determined by Gene Ontology annotation and by TargetP 1.1 server; 6 proteins from crowns and 11 proteins from leaves were localized to chloroplasts (Tables 1 and 2, Tables S1 and S4 in Supplementary data). In leaves, the frequent occurrence of differently accumulated chloroplast proteins was anticipated as they constitute the majority of the total proteins in leaves. To sustain photosynthetic reaction seems to be an important prerequisite for energy dependent cold acclimation process [7]. Both chlorophyll a-b binding proteins (gi|115793 and gi|115778; part of light-harvesting complex) decreased abundance under cold treatment. Under changing conditions, the reversible phosphorylation of chlorophyll a-b binding proteins (LHCII) represents a system for balancing the excitation energy between the photosystems I and II [46]. Therefore, the decrease of spots identified as chlorophyll a-b binding proteins seems to be a result of the reversible phosphorylation following lower level of photosynthesis in cold-treated plants. However, crowns are not photosynthetically active and they are formed by meristematic tissue where only proplastids are present; therefore, a relatively high number of proteins localised to chloroplasts in crown tissue is remarkable. Most probably, these proteins originate from proplastids. A noteworthy fact is that PsbO found in leaves and HSP70 found in crowns, both of chloroplast localisation, have revealed an excessive change in abundance which confirms an important role of chloroplast protein accumulation in the winter barley cold hardening process [17,18].

## 3. Experimental Section

### 3.1. Plant Samples

Winter barley (*Hordeum vulgare* L., cultivar Luxor) plants were cultivated as described in Jánská *et al.*[23]. In short, seedlings were grown in soil under a 12 h photoperiod and an irradiation intensity ~200 μmol m^−2^s^−1^ at a day/night temperature of 18/13 °C in a growth cabinet. In the second leaf stage, the plants were exposed for up to 3 weeks to a day/night temperature +3/2 °C and irradiation intensity was decreased to ~120 μmol m^−2^s^−1^. Following the 21 day acclimation period, a set of seedlings was also exposed to frost shock of −3 °C for 1 day. For 2D-DIGE analysis, roughly ten plants for each of all three biological replicates were used to make one sample of crown or a middle part of the second leaf tissue. Thus for each three biological replicates, at least two technical replicates (20 plant) were taken at each sampling time point (0 day—”control” samples, 1 day—”cold shock” samples, 21 day—”cold acclimation” samples and 21 day with 1 day of freezing—”frost shock” samples) from different pots in each growth cabinet.

### 3.2. Protein Extraction

Total soluble proteins were extracted as described in [47] with some modifications. Approximately 200 mg of plant material were grinded to fine powder in liquid nitrogen and then 1.5 mL of 10% TCA in acetone was added. After one hour at −20 °C, sample was centrifuged 10 min at 12,000× *g*. Resulting pellet was washed three times with cold 0.07% DTT in acetone and air-dried. Dry pellet was re-suspended in 0.5 mL of SDS buffer (30% sucrose, 2% SDS, 5% 2-mercaptoethanol, 0.1 M Tris-HCl, pH 8.0) and 0.5 mL of phenol (0.5 M Tris-HCl saturated, pH 8.0). Proteins were extracted one hour at room temperature and then centrifuged 10 min at 12,000× *g*. Upper phenol phase was placed into new microtube and proteins were precipitated with 5 volumes of cold 0.1 M ammonium acetate in methanol overnight at −20 °C. The sample was then centrifuged for 10 min at 12,000× *g* and washed three times with cold acetone, resulting pellet was air-dried. Proteins were dissolved in lysis buffer (7 M urea, 2 M thiourea, 4% *w*/*v* CHAPS, 30 mM Tris, pH 8.0) and the pH of the lysate was adjusted to 8.5 by adding 50 mM sodium hydroxide. Protein concentration was determined using 2D Quant Kit (GE Healthcare Bio-Sciences, Piscataway, NJ, USA).

### 3.3. 2D-DIGE and Image Analysis

Protein samples were labeled with Amersham CyeDye™ DIGE Fluors (GE Healthcare, Buckinghamshire, UK) according to manufacturer instructions. Covalent binding of dyes to proteins allows separation and subsequent quantitation of up to three samples in one gel. In our experiments, two samples and an internal standard (30 μg of proteins per each sample) were labeled, then mixed together and applied on a strip with an immobilized pH gradient (IPG strip, ReadyStrip™ IPG Strips, 24 cm, pH 4–7, Bio-Rad, Hercules, CA, USA) according to manufacturer instruction. Then isoelectric focusing was carried out on Protean IEF Cell (Bio-Rad, Hercules, CA, USA) with maximal voltage 10,000 V until 75,000 V-hours was reached. After isoelectric focusing, proteins in IPG strips were reduced for 12 min by 1% DTT and alkylated for 12 min by 2.5% IAA dissolved in equilibration buffer (6 M urea, 2% SDS, 20% glycerol, 0.375 M Tris-HCl, pH 8.8). Then, IPG strips were placed on the top of 12.5% polyacrylamide gels (1 mm thick) and sealed with 0.5% low melting agarose. Polyacrylamide gel electrophoresis was performed using Ettan Daltsix Electrophoresis System (GE Healthcare) for approximately 6 hours. Gels with separated proteins labeled with CyeDyes were scanned on Pharos FX Plus (Bio-Rad) at a resolution of 100 μm.

Differently accumulated proteins, *i.e.*, those that changed their abundance at least twice according to Student’s *t*-test (*p* = 0.05, three repetitions), were found using PDQuest 8.0.1 (Bio-Rad) program. The change in protein abundance in a single tissue (in leaves or in crowns) was computed as a ratio of average protein abundance at two distinct sampling dates (e.g., average abundance in leaf sample “cold shock” to average abundance in leaf “control” sample). Only the protein spots that could be detected on three gel replicates were considered for subsequent analysis.

### 3.4. Protein Identification

Spots containing differently accumulated proteins were cut out from a preparative 2D gel containing 1 mg of protein mixture and stained with Coomassie Brilliant Blue (Bio-Rad). Pieces of gel were de-stained using 100 mM NH_4_HCO_3_/ACN 1:1 (*v*/*v*) and proteins in the gel were digested for 3 h at 37 °C in 50 mM NH_4_HCO_3_ with trypsin (concentration 12.5 μg/mL, sequencing grade, Promega, Madison, WI, USA). The resulting peptides were extracted with 0.1% TFA and then desalted and concentrated using ZipTip C-18 (Millipore, Billerica, MA, USA). Peptide samples as well as external calibration standard (Bruker peptide calibration standard I, Bruker, Bremen, Germany) were mixed 1:1 with 2,5-dihydroxybenzoic acid (20 mg/mL in 33% ACN and 0.1% TFA; Sigma-Aldrich, Schnelldorf, Germany) and applied on a MALDI target plate. All mass spectrometric analysis used Biflex IV MALDI-ToF mass spectrometer (Bruker, Bremen, Germany) in reflective positive ion mode in the range of 800–4000 Da (300 laser shots for each spectrum). Spectra were analyzed in mMass 3.10.0 program (Praha, Czech Republic) [48]; signal to noise threshold was set to 3. For protein identification, the extracted peaklists were searched using Mascot server against NCBInr database (version 20120623; 18713758 sequences; 6412106995 residues) with the following settings: organism Viridiplantae—green plants; enzyme trypsin (1 misscleavage allowed), cysteine carbamidomethylation was set as a fixed modification and methionine oxidation as a variable modification; peptide tolerance was set to 0.2 Da. This settings cut off score was 72. Since the barley genome is not fully sequenced, a portion of proteins were identified on the basis of homology with proteins from different plant species. Corresponding gene identifiers on the “GeneChip Barley Genome Array” Affymetrix chip were found in the file “Barley1.na32.annot.csv” at Affymetrix’s websites [49].

### 3.5. Biological Functions of Identified Proteins

Biological functions of proteins and their cell localisation were searched in UniProtKB database [50] according to their Gene Ontology annotation (GO) [51]. To confirm cellular localisation of identified proteins with GO annotation and to determine localisation of those without GO annotation, TargetP 1.1 server [52] was used; specificity was set to >0.95.

In order to get a global overview of biological processes influenced by low temperatures, proteins were classified to categories using Web Gene Ontology Annotation Plot (WEGO) [53,54].

### 3.6. Statistical Analysis of Differently Accumulated Proteins

The differently accumulated proteins were analyzed in a R 2.15.0 environment for statistical computing [55]. In order to summarize main characteristics of data, exploratory data analysis was done using Histogram, Q-Q plot and box plot (2.698 σ). Attention was aimed at outlying observations (outlying proteins) suggested by boxplot which are those that appear to deviate markedly from other members of the data set in which they occur [56]. All differently accumulated proteins including outliers were then sorted into clusters according to their mode of accumulation using hierarchical clustering (package “stats”, function “hclust”, Ward’s method). Dendrograms were cut at height of *h* = 10 so that clusters with distinctly different modes of accumulation were created. Furthermore, the changes in protein abundance were displayed using Heat map (packages “gplots” and “RColorBrewer”, function “heatmap.2”). Since the proteins differed greatly in their abundances, all data were normalized (row centered and scaled).

## 4. Conclusions

In this study, processes of winter barley cold hardening have been glimpsed at proteome level by monitoring and comparing the response of leaves and crowns to various periods of cold and frost. It has been observed that protein and nitrogen metabolic processes were influenced by low temperatures to a similar extent in both tissues while catabolism, carbohydrate metabolism and stress response were more affected in crowns. We believe that these differences may reflect the fact that crowns are crucial for whole plant survival. Amongst the proteins which showed large changes in abundance, and therefore might play substantial roles in frost tolerant winter barley response to low temperature, AAA ATPase in leaves or HSP70 in crowns have been found. Chloroplast proteins were frequently observed as differently accumulated. Therefore, our results denote the importance of chloroplasts in low temperature response of barley. All proteins which significantly (at least twice) changed their accumulation in course of cold or frost periods were assorted to clusters according to their accumulation profile; there have been found five protein clusters in leaves and three clusters in crowns. We concluded that these clusters are likely to have a decisive influence in distinct phases of barley cold hardening as the proteins in different clusters mounted their abundance in diverse time points of low temperature exposure.

The molecular basis of winter cereals cold hardening process has been so far studied predominantly by transcriptomic methods. Generally, our proteomic study showed an accordance with previous transcriptomic studies; nevertheless, there may exist substantial differences between mRNA expression and actual protein levels due to different mechanisms of gene expression regulation. Therefore, further proteomic studies are needed in order to understand cold response regulation in cereals more profoundly.

The presented results can be considered as a starting point in selection process of frost resistance marker proteins suitable for effortless and fast crop improvement. In this perspective, it will be necessary to carry out follow-up studies to compare more barley genotypes differing in their frost resistance; from this point of view, our experiment can be regarded as a pilot study.

## Figures and Tables

**Table 1:** List of proteins up- or down- accumulated after low temperature exposure in crowns of winter barley seedlings. Plants were exposed to cold shock, cold acclimation and frost shock; differently accumulated proteins were those which changed abundance at least two times (Student’s *t*-test, *p* = 0.05, *n* = 3) in course of low temperature treatment.

Spot No 1	NCBI identifier (Probe Set ID) 2	Protein name (organism)	Score 3	*MW*_o_/pI_o_ (*MW*_t_/pI_t_) 4	SC (%)/MP 5	Biological function 6	Cluster 7	CL, O 8
***Crowns***								
2814	gi|11527563 **(Contig2786_at)**	vacuolar proton-ATPase (*Hordeum vulgare*)	187	136/5.4 (69/5.2)	37/20	ATP m. p.	1	
8408	gi|226316443	fructose-bisphosphate aldolase (*Hordeum vulgare*)	120	70/6.7 (39/6.1)	36/11	Carbohydrate m. p.	1	
3404	gi|326490940	malate dehydrogenase (*Hordeum vulgare*)	83	69/5.5 (36/5.6)	25/10	Carbohydrate m. p.	1	
1304	gi|326513418	fructokinase-2 (*Hordeum vulgare*)	139	62/5.2 (36/6.1)	39/17	Carbohydrate m. p.	1	
1311	gi|326513418	fructokinase-2 (*Hordeum vulgare*)	179	62/5.1 (36/6.1)	39/17	Carbohydrate m. p.	1	
4613	gi|357110692	6-phosphogluconate dehydrogenase, decarboxylating-like 1 (*Brachypodium distachyon*)	80	94/5.9 (53/5.6)	22/12	Carbohydrate m. p.	1	
3705	gi|326493636	enolase (*Hordeum vulgare*)	188	107/5.6 (48/5.4)	32/11	Carbohydrate m. p.	1	
2612	gi|326495636	actin (*Hordeum vulgare*)	225	89/5.4 (42/5.3)	47/21	Cytoskeleton organization	1	
108	gi|125548641	h. p. OsI_16233 *(Oryza sativa)*	85	22/4.6 (17/4.8)	47/9	Defence response	1	*
2506	gi|242074406	h. p. SORBIDRAFT_06g029250 (*Sorghum bicolor*)	84	79/5.3 (183/6.0)	11/15	Defence response	1	
12	gi|37651975	chopper chaperone (*Hordeum vulgare*)	75	20/4.6 (11/4.7)	41/6	Metal ion transport	1	
4418	gi|40317416	glutamine synthetase GSr1 (*Triticum aestivum*)	114	69/5.8 (39/5.4)	34/11	Nitrogen compound m. p.	1	
6806	gi|755762 **(HVSMEa0004F18f2_s_at)**	glutamine synthetase GS2 (*Hordeum vulgare*)	150	137/6.2 (47/5.8)	19/12	Nitrogen compound m. p.	1	c
1713	gi|326496957	thioredoxin peroxidase (*Hordeum vulgare*)	118	113/5.1 (28/6.3)	42/8	Oxidation-reduction p.	1	c
6503	gi|326492375	predicted protein (*Hordeum vulgare*)	77	85/6.2 (41/5.9)	17/7	Oxidation-reduction p.	1	
5611	gi|326493350	isocitrate dehydrogenase (*Hordeum vulgare*)	87	89/6.7 (46/6.0)	20/8	Oxidation-reduction p.	1	
7414	gi|326516152	predicted protein (*Hordeum vulgare*)	122	71/6.6 (39/6.4)	32/14	Oxidation-reduction p.	1	
5716	gi|194707130	unknown (*Zea mays*)	95	104/6.1 (40/8.5)	26/10	Protein catabolic process	1	
2901	gi|326533328	60 kDa chaperonin (*Hordeum vulgare*)	98	141/5.3 (62/5.4)	16/10	Protein folding/refolding	1	c
221	gi|113603 **(Contig86_at)**	thiol protease aleurain (*Hordeum vulgare*)	76	45/4.8 (40/6.4)	20/7	Protein m. p.	1	
4714	gi|297791831	zinc carboxypeptidase family protein (*Arabidopsis lyrata*)	79	100/5.8 (53/6.9)	22/12	Protein m. p.	1	
2008	gi|326493798	glycine rich protein, RNA binding protein (*Hordeum vulgare*)	139	18/5.4 (17/5.4)	50/10	Response to cold	1	
4304	gi|326494746	predicted protein (*Hordeum vulgare*)	148	63/5.7 (38/5.7)	37/12	Seed storage protein	1	
3601	gi|357149925	elongation factor Tu (*Brachypodium distachyon*)	148	93/5.5 (51/5.9)	40/23	Translational elongation	1	c
5506	gi|326514754	elongation factor Tu (*Hordeum vulgare*)	124	80/6.0 (48/6.0)	48/18	Translational elongation	1	
4614	gi|125580018	h. p. OsJ_36811 (Oryza sativa)	74	95/5.9 (32/6.8)	42/10	Unknown	1	
6303	gi|326491885	predicted protein (*Hordeum vulgare*)	158	61/6.2 (37/6.0)	39/14	Unknown	1	
8303	gi|326497973	predicted protein (*Hordeum vulgare*)	153	61/6.6 (39/6.3)	55/15	Unknown	1	
2808	gi|326505132	predicted protein (*Hordeum vulgare*)	105	123/5.3 (56/4.7)	18/12	Unknown	1	
4414	gi|326517467	predicted protein (*Hordeum vulgare*)	142	65/5.9 (36/5.7)	51/16	Unknown	1	
7717	gi|326517784	predicted protein (*Hordeum vulgare*)	105	104/6.6 (47/6.1)	32/14	Unknown	1	
5416	gi|356532429	LOC100792980 (*Glycine max*)	86	74/6.1 (75/7.2)	18/11	Unknown	1	
5111	gi|400094 **(Contig1675_s_at)**	JIP-23 (*Hordeum vulgare*)	84	32/6.5 (23/5.9)	38/8	Unknown	1	
2414	gi|61889374 **(Contig112_at)**	C2 domain-containing protein (*Hordeum vulgare*)	86	66/5.4 (33/5.6)	21/7	Unknown	1	
7012	gi|326493636	enolase (*Hordeum vulgare*)	79	15/6.6 (48/5.4)	32/11	Carbohydrate m. p.	2	
1402	gi|149939605	enhanced disease susceptibility 1 (*Arabidopsis lyrata*)	75	73/5.0 (70/6.1)	15/11	Defence response	2	*
4110	gi|242074406	h. p. SORBIDRAFT_06g029250 (*Sorghum bicolor*)	88	36/5.9 (183/6.0)	9/12	Defence response	2	*
5202	gi|146760217 **(Contig1508_s_at)**	beta tubulin 6 (*Hordeum vulgare*)	132	41/5.9 (51/4.8)	26/16	Microtubule-based mov.	2	
5521	gi|3915083	beta tubulin (*Cicer arietinum*)	74	78/6.1 (51/4.8)	26/12	Microtubule-based mov.	2	
704	gi|357113738	40S ribosomal protein SA-like (*Brachypodium distachyon*)	185	108/4.9 (33/4.9)	44/16	Proteosynthesis	2	
226	gi|326526175	predicted protein (*Hordeum vulgare*)	82	37/4.9 (26/6.2)	33/7	Response to cold	2	c
2001	gi|326506132	70 kDa heat shock protein (*Hordeum vulgare*)	257	15/5.2 (74/5.0)	12/8	Response to cold	2	
1705	gi|326492960	70 kDa heat shock protein (*Hordeum vulgare*)	82	102/5.1 (74/5.0)	12/8	Response to cold	2	c,*
2108	gi|326504940	predicted protein (*Hordeum vulgare*)	84	35/5.3 (26/6.2)	33/7	Response to cold	2	
817	gi|326497759	predicted protein (*Hordeum vulgare*)	86	120/4.9 (42/6.0)	28/11	Unknown	2	
5503	gi|326497759	predicted protein (*Hordeum vulgare*)	87	77/5.9 (42/6.0)	20/9	Unknown	2	
2201	gi|326494312	predicted protein (*Hordeum vulgare*)	160	39/5.3 (26/6.2)	33/7	Protein folding	3	
1004	gi|326505132	predicted protein (*Hordeum vulgare*)	175	15/5.4 (39/6.4)	32/14	Unknown	3	*

1 —spot number on 2D gel;

2 —Probe Set ID on Barley 1 Affymetrix chip;

3 —Mascot score for protein identification (cut off 72);

4 —molecular weight and pI: o—observed on 2D gel and t—theoretical;

5 —Sequence Coverage (%)/Matched Peptides;

6 —biological function according to Gene Ontology annotation;

7 —membership to cluster according to Hierarchical cluster analysis,

8 —Celullar Localisation in chloroplasts—”c” and proteins with excessive change in abundance (outliers, at least 6 fold change)—”*”.

**Table 2:** List of proteins up- or down- accumulated after low temperature exposure in leaves of winter barley seedlings. Plants were exposed to cold shock, cold acclimation and frost shock; differently accumulated proteins were those which changed abundance at least two times (Student’s *t*-test, *p* = 0.05, *n* = 3) in course of low temperature treatment.

Spot No 1	NCBI identifier (Probe Set ID) 2	Protein name (organism)	Score 3	*MW*_o_/pI_o_ (MW_t_/pI_t_) 4	SC (%)/MP 5	Biological function 6	Cluster 7	CL, O 8
***Leaves***								
6203	gi|296012105	granule-bound starch synthase I (*Solanum caripense*)	73	90/5.8 (37/6.7)	33/9	Biosynthetic process	1	
3717	gi|32400802	phosphoglycerate mutase (*Triticum aestivum*)	78	114/5.3 (30/5.4)	28/6	Carbohydrate m. p.	1	
5111	gi|303283124	pyruvate kinase (*Micromonas pusilla CCMP1545*)	76	48/5.5 (58/6.0)	21/11	Carbohydrate m. p.	1	
7837	gi|242095346	h. p. SORBIDRAFT_10g009020 (*Sorghum bicolor*)	73	101/6.0 (68/8.8)	14/9	Cell wall biogenesis	1	
5344	gi|157326682	1(10),5-germacradien-4-ol synthase (*Pinus sylvestris*)	76	34/5.7 (73/5.7)	17/11	Metabolic process	1	c
6208	gi|297791885	adenylate kinase (*Arabidopsis lyrata*)	73	90/5.8 (33/9.1)	22/6	Nucleobase-containing compound m. p.	1	c
3504	gi|22795033	putative cytochrome P450 (*Populus tremula*)	96	70/5.3 (24/7.1)	31/7	Oxidation-reduction p.	1	
2327	gi|116787193	unknown (*Picea sitchensis*)	77	43/5.2 (33/9.3)	27/9	Protein m. p.	1	c
3704	gi|75114857	ATP-dependent zinc metalloprotease FTSH 2 (*Oryza sativa Japonica*)	73	89/5.4 (73/5.5)	15/8	Protein m. p.	1	c
3429	gi|225465175	60S ribosomal protein L3-like (*Vitis vinifera*)	82	59/5.4 (25/10.0)	45/9	Proteosynthesis	1	
1307	gi|297609078	Os09g0133600 (*Oryza sativa Japonica Group*)	78	52/5.0 (27/5.1)	28/6	Response to cold	1	
8203	gi|15236846	peroxygenase 1 (*Arabidopsis thaliana*)	82	49/6.3 (28/5.8)	34/12	Response to freezing	1	
2110	gi|212724116	uncharacterised protein (*Zea mays*)	74	116/5.2 (42/9.1)	18/8	tRNA processing	1	
2111	gi|37196631	resistance protein candidate (*Helianthus annuus*)	75	19/5.1 (18/5.1)	31/7	Unknown	1	
1219	gi|296081931	unnamed protein product (*Vitis vinifera*)	74	25/4.9 (242/6.5)	5/11	Unknown	1	
4106	gi|255070529	AAA ATPase (*Micromonas sp. RCC299*)	90	117/5.4 (84/8.0)	21/14	Diverse cellular processes	2	*
5612	gi|226505792	UDP-glucose 6-dehydrogenase (*Zea mays*)	73	85/5.7 (53/6.1)	23/10	Oxidation-reduction p.	2	*
4214	gi|115778	chlorophyll a-b binding protein 1 (*Sinapis alba*)	73	128/5.5 (24/5.2)	27/7	Photosynthesis	2	c
2401	gi|115436780	33kDa oxygen evolving protein of photosystem II (*Oryza sativa Japonica Group*)	79	53/5.2 (35/6.1)	23/5	Photosynthesis	2	c,*
1521	gi|242035457	Sb01g032420 (*Sorghum bicolor*)	74	59/5.2 (80/5.6)	12/10	Transport	2	
6133	gi|145351306	F-ATPase (*Ostreococcus lucimarinus*)	75	76/5.8 (68/5.0)	17/9	ATP m. p.	3	
8202	gi|29893610	SR-rich pre-mRNA splicing activator (*Oryza sativa*)	75	143/6.0 (23/5.9)	36/7	mRNA processing	3	
529	gi|255560267	chaperonin 60 kDa (*Ricinus communis*)	96	91/4.7 (61/6.2)	25/14	Protein folding/refolding	3	
2320	gi|15236846	peroxygenase 1 (*Arabidopsis thaliana*)	83	39/5.2 (28/5.8)	30/8	Response to freezing	3	
3329	gi|255070529	AAA ATPase (*Micromonas sp. RCC299*)	74	112/5.6 (84/8.0)	12/8	Diverse cellular processes	4	*
8414	gi|255555035	ATP binding protein (*Ricinus communis*)	74	81/6.2 (75/5.8)	21/10	MAPK cascade	4	
7502	gi|297607677	kinesin (*Oryza sativa*)	75	76/6.0 (41/5.4)	33/14	Microtubule-based mov.	4	
2510	gi|755762 **(HVSMEa0004F18f2_s_at)**	glutamine synthetase GS2 (*Hordeum vulgare*)	101	66/5.2 (47/5.8)	46/10	Nitrogen compound m. p.	4	c
4233	gi|48093455	ADH-like UDP-glucose dehydrogenase (*Nicotiana tabacum*)	75	36/5.5 (42/6.2)	24/10	Oxidation-reduction p.	4	
205	gi|115793 **(Contig636_at)**	chlorophyll a-b binding protein of LHCII type III (*Hordeum vulgare*)	73	43/4.8 (29/5.0)	26/6	Photosynthesis	4	c
3410	gi|297727991	Os11g0153600 (*Oryza sativa*)	73	113/5.2 (27/10.8)	41/7	GTP catabolic process	4	c
3622	gi|226508704	elongation factor Tu (*Zea mays*)	101	75/5.3 (51/6.1)	33/15	Translational elongation	4	c
5611	gi|23476281	myb-like transcription factor 1 (*Gossypium raimondii*)	75	59/5.7 (31/7.1)	19/6	Transription	4	
2432	gi|255635003	unknown (*Glycine max*)	73	60/5.1 (34/6.4)	21/6	Unknown	4	c
5503	gi|225440886	h. p. (*Vitis vinifera*)	74	42/5.8 (18/6.0)	49/8	Unknown	4	
8537	gi|255585735	calmodulin binding protein (*Ricinus communis*)	75	78/6.2 (54/8.9)	26/13	Unknown	4	
9404	gi|225464180	h. p. (*Vitis vinifera*)	74	46/6.5 (37/6.1)	25/7	Unknown	4	
2019	gi|147785747	h. p. VITISV_033291 (*Vitis vinifera*)	81	112/4.9 (25/5.3)	38/8	Protein dephosphorylation	5	*
5630	gi|302805059	h. p. SELMODRAFT_423400 (*Selaginella moellendorffii*)	73	83/5.7 (48/8.7)	29/12	Unknown	5	*

1 —spot number on 2D gel;

2 —Probe Set ID on Barley 1 Affymetrix chip;

3 —Mascot score for protein identification (cut off 72);

4 —molecular weight and pI: o—observed on 2D gel and t—theoretical;

5 —Sequence Coverage (%)/Matched Peptides;

6 —biological function according to Gene Ontology annotation;

7 —membership to cluster according to Hierarchical cluster analysis,

8 —Celullar Localisation in chloroplasts—”c” and proteins with excessive change in abundance (outliers, at least 8 fold change)—”*”.

## Abbreviations

2D-DIGE: two dimensional difference gel electrophoresis
ACN: acetonitrile
CHAPS: 3-[(3-cholamidopropyl)dimethylammonio]-1-propanesulfonate
DHN: dehydrin
DTT: dithiothreitol
EDS1: enhanced disease susceptibility 1
GO: Gene Ontology
HSP70: 70 kDa heat shock protein
IAA: iodoacetamide
IPG: immobilised pH gradient
JIP-23: 23 kDa jasmonate-induced protein
LEA: late embryogenesis abundant
MALDI-ToF: Matrix-Assisted Laser Desorption/Ionization-Time of Flight
PsbO: 33 kDa oxygen evolving protein of photosystem II
SDS: sodium dodecyl sulfate
TCA: trichloroacetic acid
TFA: Trifluoroacetic acid
WCS: wheat cold-specific
WEGO: Web Gene Ontology Annotation Plotting
